# Unusual Dosing of Long-Acting Hydrocortisone in a Rapid Hydrocortisone Metabolizer With Addison's Disease: A Case Report

**DOI:** 10.7759/cureus.90553

**Published:** 2025-08-20

**Authors:** Nisha R Sungar, Bala Srinivasan

**Affiliations:** 1 Department of Acute Medicine, Lincoln County Hospital, Lincoln, GBR; 2 Department of Endocrinology and Diabetes, Lincoln County Hospital, Lincoln, GBR

**Keywords:** addison's disease, cortisol day curve, fast metabolizer, hydrocortisone, long-acting hydrocortisone

## Abstract

While the management of primary adrenal insufficiency often follows a structured approach with standard glucocorticoid replacement, not all patients fit into this regimen. We present the case of a woman in her 40s with Addison’s disease whose symptoms persisted despite escalating doses of immediate-release hydrocortisone (HC) and a trial of prednisolone. Cortisol day curves revealed an unusual pattern: rapid hydrocortisone clearance, with sharp declines just hours after dosing.

A trial of once-daily modified-release hydrocortisone (HCMR) (Plenadren®) yielded only modest improvement. However, further personalisation of her regimen, that is, splitting the dose into 25 mg in the morning and 5 mg in the late afternoon, led to remarkable improvement. Her symptoms stabilised, cortisol levels smoothed out, and no evidence of biochemical over-replacement was observed.

In patients with Addison’s disease and rapid hydrocortisone metabolism, standard once-daily HCMR may be insufficient. A divided dosing strategy can offer improved clinical outcomes without overtreatment. This case supports an individualised approach to steroid replacement in adrenal insufficiency.

## Introduction

Addison’s disease, or primary adrenal insufficiency, is a condition characterised by inadequate cortisol production, affecting approximately 1 in 10,000-20,000 people. Living with Addison’s disease can be challenging, especially when treatment doesn’t quite fit the patient. The goal of steroid replacement therapy is to mimic the body’s natural rhythm of cortisol production, which is highest in the early morning and gradually tapers throughout the day. For most people, this is achieved by using multiple daily doses of immediate-release hydrocortisone (HC), typically given in a standard regimen of 10 mg in the morning, 5 mg at midday, and 5 mg in the evening (10/5/5 mg), or once-daily modified-release hydrocortisone (HCMR, e.g., Plenadren®), are commonly used, often alongside fludrocortisone for mineralocorticoid replacement [1]. However, standard regimens may not be sufficient for every patient, particularly those with rapid metabolism or persistent symptoms, underscoring the need for individualised therapy.

Some patients break down hydrocortisone faster than expected. This can lead to noticeable dips in energy, symptoms of fatigue, and a significant impact on daily life, even when their prescribed doses appear to be appropriate on paper [2,3]. This comes down to how enzymes handle cortisol in the body. Two key forms of the enzyme 11β-hydroxysteroid dehydrogenase (11β-HSD) help regulate this balance: type 1 converts inactive cortisone back into active cortisol, boosting local hormone activity, while type 2 does the opposite, inactivating cortisol into cortisone and reducing its availability. This fine-tuned system means that even small differences in enzyme activity can change how much usable cortisol a patient has, helping to explain why replacement needs can vary so much between individuals [4]. These patients may find themselves struggling with ongoing symptoms despite taking their medications as advised. In fast metabolizers, rapid hepatic metabolism of hydrocortisone, partly mediated by CYP3A4, can lead to lower plasma cortisol levels within hours [1,4]. Additional factors, such as genetic polymorphisms, hepatic enzyme induction, and body composition, may further modulate cortisol metabolism, contributing to the need for individualised therapy in some patients [4].

In this report, we share the case of a woman in her 40s with Addison’s disease, whose symptoms persisted despite high doses of standard treatment. Her cortisol levels showed rapid clearance throughout the day, leaving her tired and symptomatic by afternoon. An individualised approach of splitting her long-acting hydrocortisone ( Plenadren®) dose into twice daily led to a marked improvement in her energy, symptom control, and cortisol profile.

This case highlights the importance of tailoring the therapy to the individual to provide better symptom control and improved quality of life.

## Case presentation

A woman in her 40s presented with lethargy and nausea. Laboratory investigations revealed borderline hyperkalemia (potassium 5.5 mmol/L) and mild hyponatremia (sodium 134 mmol/L) with a normal thyroid-stimulating hormone (TSH) level of 2.2 mU/L. She was diagnosed with primary adrenal insufficiency (Addison’s disease), confirmed by a short Synacthen test [1].

Over the following years, she experienced recurrent admissions with leg cramps and fatigue, during which her hydrocortisone doses were repeatedly adjusted. Six years later, she developed tremors and biochemical thyrotoxicosis, with thyroid-stimulating hormone (TSH) <0.01 mU/L, free thyroxine (FT4) 19.3 pmol/L, and free triiodothyronine (FT3) 6 pmol/L. Thyrotropin receptor antibodies (TRAb) were positive, and she was diagnosed with thyrotoxicosis--likely secondary to viral thyroiditis or primary autoimmune thyroid disease--and initiated on carbimazole 10 mg daily. Her past medical history included depression and fibromyalgia.

At the time of thyrotoxicosis diagnosis, she was taking carbimazole, fludrocortisone, and hydrocortisone 15 mg in the morning, 5 mg at midday, and 5 mg in the evening. Persistent fatigue prompted an increase in hydrocortisone to 20/10/5 mg, later adjusted to 10/10/5 mg with fludrocortisone 100 mcg daily. Cortisol day curves consistently demonstrated rapid clearance, with significant dips 2-3 hours after dosing, correlating with worsening symptoms at these times (e.g., 10:00 AM - 174 nmol/L; 12:00 PM - 49 nmol/L; 1:00 PM - 211 nmol/L; 4:00 PM - 32 nmol/L; 5:00 PM - 311 nmol/L), as evidenced in Table 1 [2,3].

**Table 1:** Showing the cortisol day curves pre and post-hydrocortisone intake It shows a significant dip in the curves before the next dose, with the patient having significant fatigue correlating at that time.

TIME	10 AM	12 PM	1 PM	4 PM	5 PM
Cortisol on Hydrocort (10 mg/10/5)	174	49	211	32	311
On Plendren® 25 mg OD	533	176	-	48	-
On Plendren® 25 mg & 5 mg	459	193	168	224	356

Cortisol day curves were performed prior to the development of thyrotoxicosis, showing a steep decline in serum cortisol from 174 nmol/L at 1 hour to <150 nmol/L by 2 hours post-dose, consistent with rapid clearance. Although thyrotoxicosis is known to accelerate glucocorticoid metabolism [4], its later development in this patient suggests that baseline pharmacokinetic variability or genetic factors were more likely to explain the early rapid metabolism [2,3].

Despite trials of prednisolone (5 mg/2.5 mg) and dehydroepiandrosterone (DHEA) (50 mg), symptoms of fatigue affecting her daily life persisted. Multi-disciplinary team (MDT) discussions supported a long-acting hydrocortisone, but due to a lack of formulary inclusion, prednisolone was initially attempted, without improvement. She was offered continuous subcutaneous hydrocortisone infusion (CSHI) via a pump, which has shown improved health-related quality of life in patients with adrenal insufficiency [5], but she declined as it would not be compatible with her lifestyle. An individual funding request was subsequently submitted to the local formulary committee, permitting a trial of long-acting hydrocortisone (cost approximately £480 vs. £2 for conventional HC) [6].

**Figure 1:**
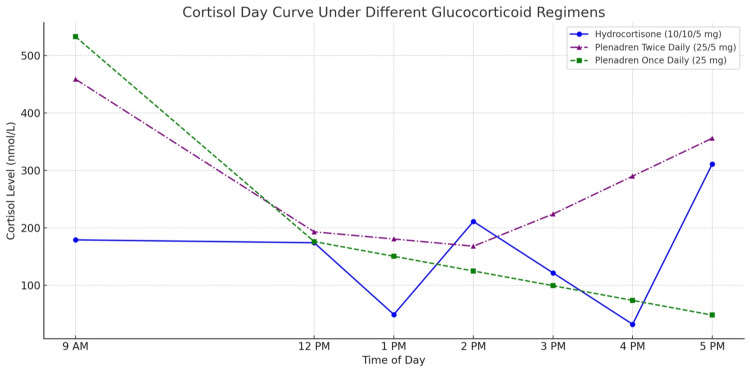
Cortisol day curve at the start and showing the dips before the next dose, correlating clinically with her poor symptom control • Hydrocortisone (10/10/5 mg) in blue • Plenadren once daily (25 mg) in green • Plenadren twice daily (25/5 mg) in purple

Long-acting hydrocortisone was later introduced at 25 mg once daily. It showed modest improvement during the day. However, she continued to experience afternoon fatigue, showing a dip in cortisol curves in the evening. This prompted us to question whether the standard once-daily regimen was sufficient. We then split the dose into 25 mg AM and 5 mg PM, which resulted in noticeable symptomatic improvement and smoother cortisol levels (Table 1 and Figure 1). A 24-hour urinary free cortisol was done, which was within normal limits--62 nmol/24 hr (normal=0-199 nmol/24 hr), ruling out over-replacement.

She completed 18 months of carbimazole and was in remission, but relapsed three months later. Treatment options, including long-term carbimazole, surgery, or radioactive iodine, were discussed. She subsequently underwent total thyroidectomy for relapsed thyrotoxicosis and developed postoperative hypoparathyroidism, which is currently managed with alfacalcidol.

## Discussion

Primary adrenal insufficiency (PAI), also known as Addison’s disease, is an endocrine disorder characterised by inadequate production of glucocorticoids (cortisol) and often mineralocorticoids (aldosterone) due to adrenal cortex destruction and affecting approximately 1 in 10,000-20,000 people [4]. Recent registry-based data estimate a prevalence of approximately 100-140 cases per million in Western populations. The clinical presentation is often nonspecific and includes fatigue, weight loss, hypotension, salt craving, hyperpigmentation, nausea, and abdominal pain [4]. Diagnosis is typically confirmed with a short synacthen test, where a peak serum cortisol of <500 nmol/L at 30 or 60 minutes post 250 mcg synthetic adrenocorticotropic hormone (ACTH) is suggestive of adrenal insufficiency [4].

**Figure 2:**
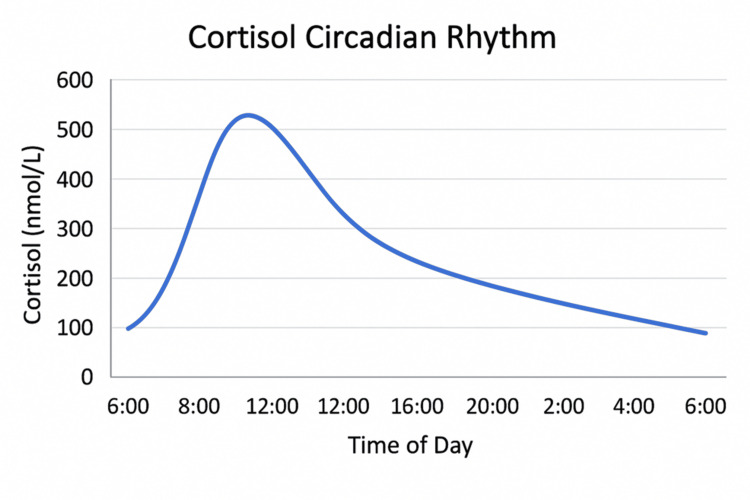
Normal cortisol day curve

Cortisol in healthy individuals follows a natural circadian rhythm where it peaks early in the morning (between 6 and 8 AM), declines throughout the day, and reaches a nadir during the late evening and early night (Figure 2) [2]. The goal of replacement therapy in Addison’s disease is to closely replicate this pattern to avoid symptoms of both under- and over-replacement and to improve metabolic outcomes [1,2]. This rhythm is essential for energy balance, immune function, and metabolic regulation. In fast metabolizers, rapid hepatic metabolism of hydrocortisone, partly mediated by CYP3A4, can lead to lower plasma cortisol levels within hours [1,4,7]. Additional factors, such as genetic polymorphisms, hepatic enzyme induction, and body composition, may further modulate cortisol metabolism, contributing to the need for individualised therapy in some patients [4,7]. Higher body surface area or lean body mass may increase volume of distribution and metabolic clearance [2]. Liver enzyme activity, particularly in hypermetabolic states (e.g. hyperthyroidism), can influence metabolism [4]. Lastly, drug-drug interactions like medications that induce CYP3A4 (e.g., some anticonvulsants, rifampin) increase glucocorticoid metabolism [1]. Thyrotoxicosis, like in our case, may also accelerate glucocorticoid clearance via hepatic enzyme induction [4]. Fast hydrocortisone metabolism may also be influenced by several factors, including genetic polymorphisms in enzymes like 11β-hydroxysteroid dehydrogenase type 1 or cytochrome P450 isoforms, gastrointestinal absorption variability, and hepatic enzyme induction. While such mechanisms remain incompletely understood, their clinical implications are significant, often resulting in symptom recurrence despite appropriate dosing [4,7].

Traditional glucocorticoid replacement therapy uses immediate-release hydrocortisone (HC), administered two or three times daily in divided doses. Pharmacokinetic studies have shown that this regimen often produces supraphysiological cortisol levels shortly after each dose and suboptimal levels later in the day [2] (modelling and cortisol day-curve studies), which may contribute to persistent symptoms despite apparent adherence [3] (clinical outcome study demonstrating variable symptom control). HCMR, such as Plenadren®, was developed to provide a once-daily regimen that more closely mimics physiological cortisol secretion and reduces total cortisol exposure [1,2]. It provides an initial quick release followed by a slower release of cortisol throughout the day, better reflecting the body's natural rhythm [3]. Rapid hepatic metabolism and genetic variability are recognised contributors to inter-individual differences in hydrocortisone clearance. Mechanistic and pharmacokinetic studies, such as those by Isidori et al. [7], demonstrate altered cortisol exposure-time profiles linked to hepatic enzyme activity. 

The DREAM trial demonstrated that once-daily HCMR reduced weight gain and improved glucose metabolism and innate immunity compared to standard therapy [7]. Another study by Johannsson et al. showed improved cortisol exposure-time profile and health-related quality of life in patients treated with HCMR compared to conventional regimens [1].

However, inter-individual variability in hydrocortisone metabolism poses a significant challenge. Rapid drops in cortisol within two to three hours of dosing can result in daily symptomatic troughs, as seen in this case [4]. These dips are not always captured by random serum cortisol levels--day curves are necessary but not widely used in every unit [1]. Some patients, such as the one presented in this case, demonstrated rapid cortisol clearance, which was confirmed by serial cortisol day curves. These patients may fail to achieve adequate cortisol exposure despite adherence and dose escalation.

In our patient, cortisol levels dropped significantly within 2-3 hours of HC dosing (e.g., 12 pm: 49 nmol/L; 4 pm: 32 nmol/L), indicating insufficient coverage. Even switching to prednisolone (a longer-acting steroid) did not improve symptoms. After a successful Individual Funding Request, she was started on HCMR (Plenadren®), initially once daily. This showed modest improvement, but symptoms recurred in the afternoon. Splitting the dose to 25 mg AM and 5 mg PM led to significant improvement in fatigue and a more stable cortisol curve, without signs of over-replacement, as confirmed by a normal 24-hour urinary free cortisol level (62 nmol/24 hr; normal range: 0-199 nmol/24 hr).

Though Plenadren® is licensed for once-daily use, emerging evidence suggests that twice-daily HCMR may benefit a subset of patients with faster metabolism or persistent afternoon symptoms [3]. While reference ranges for cortisol levels with Plenadren® are lacking, clinical improvement and biochemical monitoring (such as day curves and urinary cortisol) guide dose adjustments [1-3]. No standard cortisol targets exist for modified-release formulations, making interpretation of lab values tricky [1,2]. A 24-hour urinary free cortisol may miss diurnal variation or peaks/troughs that correlate better with symptoms [1,8].

This case reinforces the principle of individualised therapy in adrenal insufficiency. Standard regimens may not be suitable for every patient, and therapeutic flexibility, including off-label dosing frequencies, can significantly enhance quality of life. Further studies are needed to better identify patients with rapid metabolism and to explore optimal monitoring strategies in HCMR therapy [7-9]. In such cases, patient-reported outcome measures (PROMs) offer valuable insights into symptom burden and quality of life. Tools like the AddiQoL questionnaire specifically assess fatigue, energy levels, and emotional well-being in patients with adrenal insufficiency. Similarly, the Patient-Reported Outcomes Measurement Information System (PROMIS) Fatigue scale is a validated measure for fatigue-related impact on daily function. Incorporating PROMs into routine clinical practice enables more patient-centred care and allows for better evaluation of treatment efficacy beyond biochemical parameters [10]. Although PROMs were not formally scored in this case, symptom improvement following the dose adjustment was patient-reported and clinically evident in the cortisol day curve.

## Conclusions

This case demonstrates the need for personalised approaches in managing primary adrenal insufficiency, particularly in patients with atypical pharmacokinetics. While once-daily HCMR (Plenadren®) is approved and generally effective, it may not provide adequate symptom control in individuals with rapid hydrocortisone metabolism. Our patient experienced significant clinical improvement and more stable cortisol levels after transitioning to a split-dose regimen of Plenadren®. This strategy allowed symptom resolution without evidence of glucocorticoid over-replacement, as confirmed by both clinical evaluation and biochemical monitoring.

The standard "one-regimen-fits-all" approach may not serve all patients with Addison’s disease. This case reinforces the importance of recognising individual variability in glucocorticoid metabolism and tailoring replacement therapy accordingly. Cortisol day curves and patient-reported outcomes were crucial in guiding management. Further studies are warranted to understand predictors of fast metabolism and to evaluate long-term outcomes of modified dosing strategies for these sub-groups.
